# Isolation and Preliminary Characterization of Salt-Tolerant Polyhydroxyalkanoate-Producing Bacteria from the Hon Khoi Saltern, Khanh Hoa, Vietnam

**DOI:** 10.3390/microorganisms14040825

**Published:** 2026-04-03

**Authors:** Thoa Kim Nguyen, Nhung Thi Hong Lai, Minh Thi Tuyet Phan, Tu Thi Minh Hoa, Duc Quan Nguyen

**Affiliations:** 1 Institute of Biology, Vietnam Academy of Science and Technology, 18 Hoang Quoc Viet, Nghia Do, Hanoi 11300, Vietnam; hanhunglai@gmail.com (N.T.H.L.); phanthituyetminh1973@gmail.com (M.T.T.P.); hoathiminhtu@gmail.com (T.T.M.H.); ducquan0709@gmail.com (D.Q.N.); 2 Vietnam Academy of Science and Technology, Graduate University of Science and Technology, 18 Hoang Quoc Viet, Nghia Do, Hanoi 10072, Vietnam

**Keywords:** polyhydroxyalkanoate, saltern, salt-tolerant bacteria, *Salinivibrio* sp., *Halomonas* sp., *Priestia* sp., *Bacillus* sp.

## Abstract

Polyhydroxyalkanoates (PHAs) are biodegradable microbial polyesters that represent a promising sustainable alternative to petroleum-based plastics. Salterns, hypersaline environments, are recognized as significant sources of halotolerant microorganisms that can produce PHAs in high-salinity conditions; however, Vietnamese saltern ecosystems have not been extensively investigated. This research aimed to isolate and initially characterize salt-tolerant bacteria capable of synthesizing PHAs from the Hon Khoi saltern in Khanh Hoa Province, Vietnam. A total of 37 halotolerant bacterial isolates were obtained, and potential PHA-producing strains were initially screened using Sudan Black B and Nile Blue A. TEM microscopy was then employed to confirm the existence of PHA granules. Furthermore, FTIR spectroscopy and GC–MS/MS spectrometry were utilized to analyze the chemical structure and monomer composition of the extracted polymers. Six isolates were identified as PHA-producing bacteria, including *Salinivibrio* sp. HK101 and HK116, *Halomonas* sp. HK105, *Priestia* sp. HK125 and HK142, and *Bacillus* sp. HK130. These strains exhibited growth across 3–10% NaCl and temperatures from 25 to 45 °C. *Priestia* sp. HK142 and *Salinivibrio* sp. HK101 exhibited the most substantial PHA accumulation, achieving 50.72 ± 1.83% and 42.07 ± 1.8% of DCW, respectively. These results indicate that the Hon Khoi saltern represents a promising source of halotolerant PHA-producing bacteria with potential relevance for future biopolymer production studies.

## 1. Introduction

Polyhydroxyalkanoates (PHAs) are biodegradable polymers synthesized by numerous Gram-positive and Gram-negative bacteria, representing a promising sustainable alternative to traditional petrochemical-based plastics [1]. PHAs are primarily classified according to the carbon chain length of their monomeric units, including short-chain-length PHAs (scl-PHAs; C3–C5), medium-chain-length PHAs (mcl-PHAs; C6–C14), and long-chain-length PHAs (lcl-PHAs; ≥C15) [2]. The process of PHA accumulation is believed to serve as an adaptive strategy that allows microorganisms to survive under extreme conditions, including nutrient deficiency and high levels of salinity [3]. These polyesters accumulate as intracellular granules, and their composition, physicochemical characteristics and thermal properties are strongly influenced by both the producing strain as well as cultivation conditions [2,4]. Among PHAs, poly(3-hydroxybutyrate) (PHB), poly(3-hydroxybutyrate-co-3-hydroxyvalerate) (PHBV), and their copolymers demonstrate favorable properties, including biodegradability and biocompatibility, making them suitable for a wide range of applications in agriculture, medicine and industry, such as seed coating, food packaging, 3D printing, tissue engineering, drug delivery systems, and wound healing [5,6].

Growing environmental concerns associated with petroleum-based plastics have intensified global interest in bioprospecting for novel microorganisms capable of producing PHAs efficiently [4,7]. This has led to extensive research into diverse and extreme environments as potential sources for novel PHA-producing microbial strains [8]. Salterns represent an extreme and dynamic habitat characterized by high salinity (sea water: 3–4%; intermediate pond: 10–25%; crystallizer pond: 30–37%), strong osmotic stress, and pronounced temperature fluctuations (25–55 °C) [9,10,11]. These environmental pressures promote the proliferation of microorganisms that synthesize PHAs as stress-response polymers, thereby enhancing the probability of isolating efficient PHA producers with potentially advantageous characteristics [1]. Simultaneously, saltern ecosystems frequently display spatial and temporal salinity gradients, thereby promoting high microbial diversity and the development of specialized microbial taxa with distinctive metabolic functions [12].

In this context, salt-tolerant (halotolerant) and halophilic bacteria have attracted increasing attention as promising microbial bioreactors for the production of PHA in high-salinity conditions. To date, approximately 150 bacterial genera have been reported to synthesize and accumulate intracellular carbon and energy storage polymers [13]. Haloarchaea, including *Halococcus* spp., *Haloferax* spp., *Natronococcus* spp., and *Natronobacterium* spp., thrive at extremely high salinities (100–300 g/L NaCl) and are recognized for their capacity to synthesize PHA biopolyesters [1,14]. Halotolerant bacteria present several advantages for PHA production, principally by reducing the risk of microbial contamination and lowering production costs. These advantages arise from the feasibility of continuous fermentation under high-salinity conditions, thereby minimizing sterilization demands, freshwater usage, and energy consumption, while also facilitating more efficient downstream processing, including cell disruption and downstream polymer recovery [6,15].

Despite their environmental advantages and potential, the extensive industrialization of microbial PHAs encounters considerable obstacles. These challenges primarily stem from inconsistent material quality, suboptimal thermal and mechanical properties, PHA degradation, scalability issues, high production costs, and market competition with petroleum-based plastics [16]. Addressing these limitations necessitates ongoing research into identifying and developing novel microbial strains, especially those originating from underexplored extreme environments like hypersaline habitats. Microorganisms acclimated to such extreme environments may facilitate the discovery of PHA producers with enhanced productivity and improved polymer properties [17,18]. Previous studies have successfully isolated PHA-producing bacteria from saltern environments, including members of the genera *Halomonas*, *Bacillus*, *Pseudomonas, Staphylococcus, Chromohalobacter*, and *Alcaligenes*, which were shown to accumulate PHB and PHBV under saline conditions [1,2,19,20]. Saltern ecosystems in Vietnam remain underexplored with respect to the identification and characterization of halotolerant PHA-producing microorganisms, highlighting the need for further investigation. The aim of this study was to isolate, as well as conduct the preliminary characterization of, salt-tolerant bacteria capable of producing PHAs from the Hon Khoi saltern in Khanh Hoa, Vietnam. Potential PHA-producing isolates were initially screened for PHA accumulation, followed by ultrastructural analysis of intracellular inclusion bodies using transmission electron microscopy (TEM). Selected isolates were further identified and characterized, and their PHA content and monomer composition were analyzed using Gas Chromatography–Tandem Mass Spectrometry (GC–MS/MS). The chemical structure of the extracted polymers was verified through Fourier transform infrared spectroscopy (FTIR). The findings of this study are expected to contribute to future studies on the optimization and application of isolated bacterial strains for the sustainable production of PHAs.

## 2. Materials and Methods

### 2.1. Sample Collection and Isolation of Bacteria

Saline soil and sediment samples were collected from areas surrounding the salt pans of the Hon Khoi saltern, Khanh Hoa Province, Vietnam (approximately 12.539116° N, 109.206517° E). Samples were aseptically collected into sterile 50 mL Falcon tubes and transported to the laboratory for further processing. The collected samples were pooled and homogenized by gentle manual swirling in a sterile Erlenmeyer flask prior to bacterial isolation. For isolation, 10 g of the homogenized saline soil–sediment sample was suspended in 90 mL of sterile saline solution containing 3% (*w*/*v*) NaCl and vigorously mixed. The resulting suspension was serially diluted using sterile saline solution supplemented with 3% (*w*/*v*). Aliquots (500 µL) from dilution levels 10^−3^ to 10^−5^ were spread onto high-salt Luria–Bertani (LB) agar plates adjusted to a final NaCl concentration of 3% (*w*/*v*) NaCl. The plates were incubated at 37 °C for 48 h. After incubation, morphologically distinct colonies were selected and purified by repeated streaking on high-salt LB agar until pure cultures were obtained. The purified isolates were maintained on the same medium for subsequent screening and characterization.

### 2.2. Primary Screening for PHA Accumulation

Primary screening of potential PHA-producing isolates was conducted using Sudan Black B plate staining, with Nile Blue A fluorescence staining employed as a complementary cellular-level assay. For Sudan Black B staining, bacterial colonies grown on high-salt LB agar were flooded with Sudan Black B solution directly on the agar surface and incubated for 30 min. Excess stain was removed, and the plates were gently rinsed with 70% (*v*/*v*) ethanol until the background agar became clear. Colonies exhibiting dark blue to black coloration after destaining were considered potential PHA producers.

Nile Blue A staining was employed to visualize intracellular lipid inclusions at the cellular level. Briefly, bacterial cells were stained with a Nile Blue A solution and examined using an ECLIPSE Ti fluorescence microscope (Nikon, Tokyo, Japan) with an excitation wavelength of 460 nm. Cells exhibiting orange fluorescence were considered indicative of intracellular polyester inclusions. Isolates showing positive responses in both screening assays were selected for further analysis [21].

### 2.3. Transmission Electron Microscopy

TEM was used to examine the ultrastructural features of bacterial cells and to visualize intracellular inclusion bodies. Representative isolates that tested positive in the primary screening were selected for TEM analysis. Cell samples were prepared according to standard TEM procedures, and ultrathin sections were examined using a JEOL JEM-1010 transmission electron microscope (JEOL Ltd., Tokyo, Japan) to assess the presence and intracellular distribution of inclusion bodies in the cytoplasm [22,23].

### 2.4. Identification and Characterization of PHA-Producing Isolates

PHA-positive isolates were subjected to a series of analyses, including morphological, physiological, biochemical, and molecular analyses, to facilitate their identification and characterization.

Morphological characteristics of the selected isolates were examined after cultivation on high-salt LB agar, including colony shape, size, margin, elevation, surface texture, opacity, pigmentation, and diameter. Cell morphology, endospore formation, and Gram reaction were examined by Olympus CH-2 light microscopy using a 100× oil-immersion objective (Olympus, Tokyo, Japan) following conventional Gram staining. Motility was assessed using semi-solid agar medium (0.3% agar).

Physiological and biochemical characterization of the selected isolates was performed using standard microbiological assays. Oxidase activity was determined using oxidase reagent, and catalase activity was evaluated by observing bubble formation following the addition of 3% (*v*/*v*) hydrogen peroxide. The Voges–Proskauer (VP) test was conducted to assess acetoin production according to standard protocols [24].

The effects of temperature on bacterial growth were evaluated by incubating the isolates at 25, 30, 37, 40, and 45 °C. Salt tolerance was assessed by culturing the isolates in LB-based media containing 0%, 3%, 5%, 10%, and 15% (*w*/*v*) NaCl. For the 0% NaCl condition, the medium consisted only of Bacto tryptone and yeast extract without NaCl, whereas for the other conditions, NaCl was added to reach the indicated concentrations. Bacterial growth under these conditions was monitored for 48 h and qualitatively assessed based on visible turbidity, recorded as positive (+), weakly positive (±), or negative (−).

Carbon source utilization was examined in Phenol Red broth base (HiMedia, Thane, India) as the basal medium, supplemented with D-glucose, sucrose, maltose, D-galactose, L-arabinose, D-xylose, D-mannose, and D-mannitol as sole carbon sources. Hydrolytic activities were evaluated using soluble starch, casein, and carboxymethyl cellulose (CMC). Growth and enzymatic activities were interpreted based on visible growth or clear zone formation and recorded qualitatively as positive (+), weakly positive (±), or negative (−).

For molecular identification, genomic DNA was extracted from the selected isolates using a Genomic DNA Purification Kit (Thermo Fisher Scientific, Waltham, MA, USA). The 16S rRNA gene was amplified by PCR using the universal bacterial primers 27F (5′-AGAGTTTGATCCTGGCTCAG-3′) and 1492R (5′-TACGGYTACCTTGTTACGACTT-3′) [25]. PCR products were purified and sequenced by 1st Base (Singapore). The obtained sequences were compared with reference sequences available (NR_157685.1, NR_041552.1, NR_115009.1, NR_043536.1, NR_178426.1, NR_027590.1, NR_114030.1, NR_180388.1, NR_115697.1, NR_116945.1, NR_114161.1, NR_115605.1, NR_115953.1, NR_116873.1, NR_125615.1, NR_074923.1, NR_118996.1, NR_113265.1, and NR_117946.1) in the NCBI GenBank database using the BLAST algorithm. Phylogenetic analysis was performed using the neighbor-joining method implemented in MEGA X, with bootstrap analysis based on 1000 replicates.

### 2.5. Fermentation of PHA

A single isolated colony of the selected strain was inoculated into a test tube containing 3 mL of high-salt LB broth supplemented with 3% (*w*/*v*) NaCl and incubated at 37 °C with shaking at 200 rpm for 24 h to obtain the first-step preculture (seed 1). Subsequently, a second-step seed was prepared by transferring 1 mL of seed 1 into a 100 mL Erlenmeyer flask containing 19 mL of high-salt LB broth, followed by incubation at 37 °C and 200 rpm for 18 h (seed 2).

For PHA production, 5% (*v*/*v*) of seed 2 was inoculated into a 1 L Erlenmeyer flask containing 300 mL of sterile saline production medium composed of (g/L): carbon source, 30; peptone, 4; Na_2_MoO_4_·2H_2_O, 0.005; MnSO_4_, 0.005; FeSO_4_·7H_2_O, 0.01; CaCl_2_·2H_2_O, 0.02; NaCl, 30; MgSO_4_·7H_2_O, 0.2; KH_2_PO_4_·2H_2_O, 0.2; and K_2_HPO_4_, 1.6. The medium was adjusted to pH 7.0 before sterilization.

The carbon source was supplied as glycerol, glucose, sucrose, or a mixed carbon strategy in which glucose (20 g/L) and 1,4-butanediol (9 g/L) were provided at the beginning of cultivation, followed by the addition of valeric acid (1 g/L) after 24 h of fermentation. Fermentation was carried out at 37 °C and 200 rpm for 72 h. At the end of cultivation, the culture broth was centrifuged at 8000 rpm for 10 min to harvest the cell biomass, and the supernatant was removed.

Based on the carbon source screening results, the most suitable carbon source for each strain was selected for further optimization. The effect of the carbon-to-nitrogen (C/N) ratio on growth and PHA production was subsequently investigated using peptone as the nitrogen source, by adjusting the initial C/N ratios of the production medium to 1:20, 1:25, 1:30, and 1:35 (mol/mol), while maintaining all other cultivation conditions unchanged.

### 2.6. Determination of Dry Cell Weight

The cell pellet was washed twice with distilled water, dried at 100 °C to constant weight, and the dry cell weight (DCW) was expressed as g/L.

### 2.7. Extraction and Purification of PHA

PHA was extracted and purified from dried cell biomass using a modified version of the method described by [26]. Briefly, the dried biomass was treated with a mixture of sodium hypochlorite and chloroform at a 1:35 (*w*/*v*) ratio and incubated at 60 °C for two hours under gentle agitation (100 rpm). After incubation, the aqueous phase containing cell debris was discarded, and the chloroform phase containing the dissolved PHA was transferred into a glass Petri dish. PHA was precipitated by the addition of a methanol–water mixture (7:3, *v*/*v*), followed by centrifugation at 12,000 rpm for 15 min. The resulting polymer pellet was washed twice with 95% ethanol and air-dried overnight at room temperature before further analysis.

### 2.8. Determination of PHA Content and Monomer Composition

PHA content (%) was calculated as the percentage of purified PHA relative to DCW, as described in Section 2.6.

The quantitative monomer composition of PHA, expressed as the molar fractions (%mol) of 3-hydroxybutyrate (3HB) and 3-hydroxyvalerate (3HV), was determined by acidic methanolysis followed by GC–MS/MS analysis. Dried cell biomass (0.1000 ± 0.001 g) was transferred into a 10 mL screw-cap glass tube, to which 0.2 mL of toluene and 1.5 mL of HCl/methanol (85:15, *v*/*v*) were added. The mixture was tightly sealed and methanolized in a thermostatic water bath at 100 °C for 2 h. After cooling to room temperature, 1–2 mL of hexane was added, vortexed thoroughly, and centrifuged at 7000 rpm for 10 min. The upper organic phase was collected, filtered through a 0.45 µm solvent-resistant membrane, and transferred into GC vials.

GC–MS/MS analysis was performed using a TRACE 1300 gas chromatograph coupled with a TSQ 8000 Evo triple quadrupole mass spectrometer (Thermo Fisher Scientific, USA) equipped with an HP-5ms Ultra Inert capillary column (30 m × 0.25 mm × 0.25 µm, Agilent, Santa Clara, CA, USA). The injector temperature was set at 230 °C and operated in splitless mode, with a splitless time of 1.0 min. Helium as the carrier gas at a constant flow rate of 1.5 mL/min. The oven temperature program was as follows: an initial temperature of 40 °C (held for 2 min), increased at 8 °C/min to 280 °C, and held for 5 min. Mass spectrometric detection was carried out in electron ionization (EI) mode, with a transfer line temperature of 280 °C and an ion source temperature of 230 °C. Full-scan mass spectra were acquired over an *m*/*z* range of 33–500 with a solvent delay of 3.2 min.

Monomer identification was performed by comparing retention times and mass spectra with PHB and PHBV (8 mol% 3HV) standards (Sigma-Aldrich, St. Louis, MO, USA) and the NIST 19 mass spectral library. Quantification of 3HB was conducted using an external calibration curve constructed from a PHB standard subjected to the same methanolysis procedure. The molar fraction of 3HV was calculated based on the relative peak areas of 3HB and 3HV in the GC chromatograms.

### 2.9. Structural Characterization of PHA

FTIR spectroscopy was employed to characterize the chemical structure of PHA and to identify its characteristic functional groups. FTIR spectra were recorded using a Thermo Nicolet NEXUS 670 FTIR spectrometer (Thermo Scientific, USA) over the wavenumber range of 4000–500 cm^−1^, with particular emphasis on the ester carbonyl stretching vibration at 1700–1738 cm^−1^ and alkyl (C–H) stretching vibrations at 2923–2975 cm^−1^, typical of PHA [27].

Proton nuclear magnetic resonance (^1^H-NMR) spectroscopy was used to confirm the polymer structure further and to identify characteristic proton signals associated with 3HB and 3HV units. ^1^H-NMR spectra were acquired using a Bruker AVANCE NEO 600 MHz NMR spectrometer (Bruker, Rheinstetten, Germany).

### 2.10. Statistical Analysis

Experimental data were statistically analyzed using Student’s *t*-test in Microsoft Excel (Microsoft Corp., Redmond, WA, USA). All experiments were performed at least in triplicate, and the results are expressed as mean ± standard deviation (SD). Graphs were generated using OriginPro 8.5.1 (OriginLab Corporation, Northampton, MA, USA).

## 3. Results

### 3.1. Isolation and Primary Screening of Halotolerant PHA-Producing Bacteria from the Hon Khoi Salt Fields

**Figure 1:**
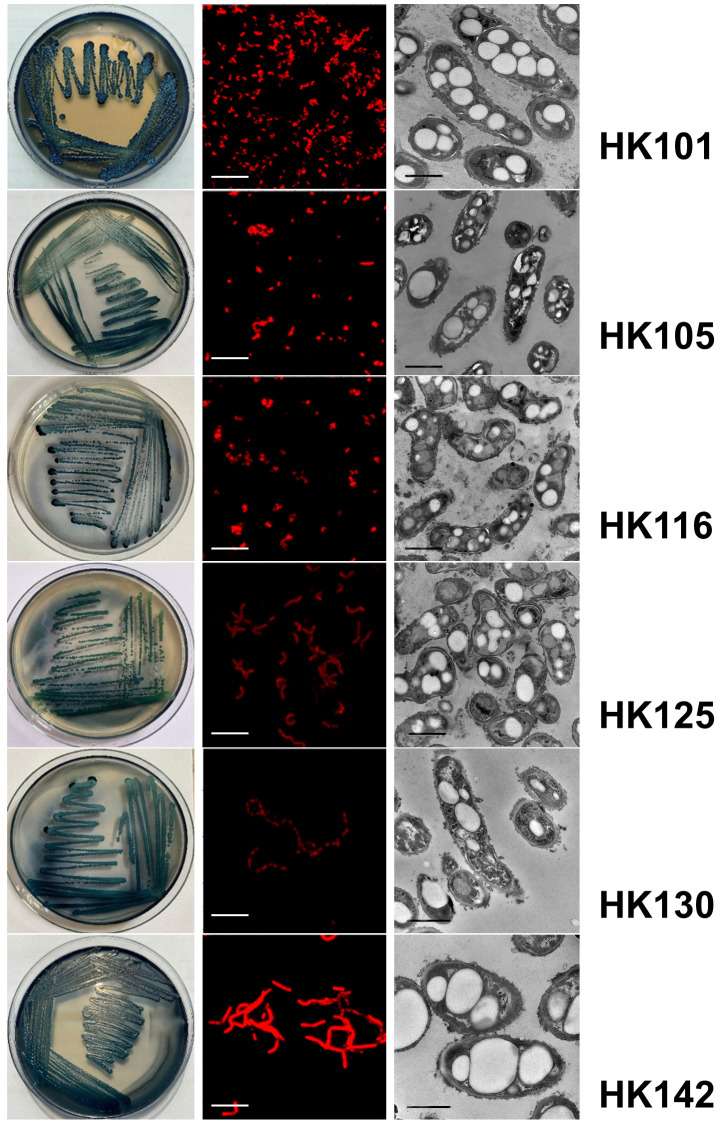
Primary screening and confirmation of intracellular PHA accumulation in halotolerant bacterial isolates from the Hon Khoi salt fields. From top to bottom: HK101, HK105, HK116, HK125, HK130, and HK142. The left column shows Sudan Black B staining on agar plates, the middle column presents Nile Blue A fluorescence images indicating intracellular polyester accumulation, and the right column displays transmission electron microscopy images confirming the presence of intracellular inclusion bodies within the cytoplasm. Scale bar = 1 µm (fluorescence images) and 1 µm (TEM images).

A total of 37 halotolerant bacterial isolates were obtained from salt field samples collected at the Hon Khoi salt pans. These isolates were initially screened for intracellular polyester accumulation using Sudan Black B and Nile Blue A staining (Figure 1). Among them, six isolates (HK101, HK105, HK116, HK125, HK130, and HK142) exhibited positive staining responses, characterized by dark intracellular granules in Sudan Black B staining and orange fluorescence under Nile Blue A staining, indicating the presence of intracellular polyester inclusions.

To further confirm intracellular inclusion body formation, the six positively screened isolates were subjected to TEM analysis. TEM observations revealed distinct electron-dense inclusion bodies in the cytoplasm, consistent with intracellular PHA granules. Based on these combined screening and confirmation results, the six isolates were identified as potential PHA-producing halotolerant bacteria and were selected for subsequent characterization and fermentation studies (Figure 1).

### 3.2. Characterization and Identification of Selected Strains

The morphological, physiological, and biochemical characteristics of the six selected isolates (HK101, HK105, HK116, HK125, HK130, and HK142) are summarized in Table 1. Phenotypic diversity was observed among the isolates in terms of colony morphology, cellular characteristics, physiological tolerance, and metabolic capabilities. Colony morphology varied markedly, with HK101, HK105, and HK116 forming round, smooth, convex, and opaque colonies with cream to yellow pigmentation and colony sizes ranging from approximately 0.5 to 2.5 mm. In contrast, HK125, HK130, and HK142 produced round colonies with irregular surfaces or undulate margins, opaque appearance, and creamy white to grayish-white coloration, with colony diameters of approximately 2.0–3.0 mm. HK101 and HK116 consisted of curved rod-shaped cells, whereas HK105, HK125, HK130, and HK142 displayed straight rod morphology. Despite these morphological differences, all six isolates were motile. Further differentiation of the isolates was evident based on Gram-staining characteristics and endospore formation. Gram staining and endosperm formation differentiated the isolates into two distinct groups: HK101, HK105, and HK116 were Gram-negative and non-spore-forming bacterial strains, whereas HK125, HK130, and HK142 were Gram-positive and endospore-forming bacterial strains.

All isolates exhibited halotolerant behavior, with growth observed at 3–5% NaCl (Table 1). Differences in salt tolerance were observed at higher salinity levels. HK105 and HK125 displayed the highest salt tolerance, with sustained growth observed at NaCl concentrations up to 15%, whereas the remaining isolates showed limited growth at higher NaCl concentrations. All six isolates grew over a broad temperature range, with consistent growth observed between 25 and 40 °C. Growth at 45 °C varied among strains, with HK105 showing no growth at this temperature.

Biochemical and metabolic profiles further distinguished the isolates. All isolates were positive for catalase activity, while oxidase and Voges–Proskauer reactions varied among strains. Voges–Proskauer reactions were negative in HK105 and HK142, while positive reactions were detected in HK101, HK116, HK125, and HK130. Carbohydrate utilization profiles showed both common and strain-specific patterns. All isolates were able to utilize D-glucose and maltose as carbon sources. Sucrose was the second most frequently utilized carbon source, being positive in all isolates except HK105. Utilization of D-galactose, L-arabinose, D-xylose, D-mannose, and D-mannitol varied among the assessed strains. Differences were also noted in hydrolytic activities toward soluble starch, casein, and carboxymethyl cellulose. Soluble starch and CMC were able to be hydrolyzed by HK101, HK116, HK125, HK130, and HK142; casein hydrolysis was observed in HK101, HK125, HK130, and HK142. In contrast, HK105 did not exhibit hydrolytic activity toward soluble starch, casein, or CMC. Collectively, these phenotypic characteristics support the taxonomic differentiation of the selected isolates and complement the molecular identification results.

**Figure 2:**
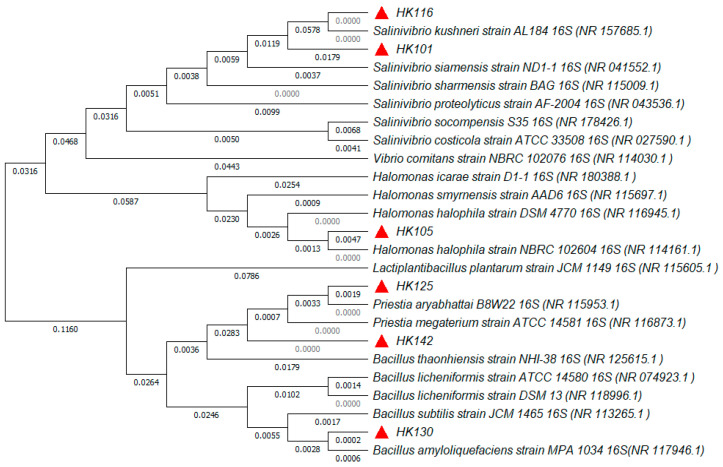
Phylogenetic tree based on 16S rRNA gene sequences showing the taxonomic positions of the six isolates (HK101, HK105, HK116, HK125, HK130, and HK142) in relation to closely related reference strains. The tree was constructed using the neighbor-joining method. Reference sequences were retrieved from the NCBI GenBank database, and accession numbers are given in parentheses. Numbers at branch nodes indicate evolutionary distances (substitutions per nucleotide position). The isolates obtained in this study are marked with red triangles.

To further clarify the taxonomic positions of the six isolates, a phylogenetic analysis of 16S rRNA gene sequences was performed. The resulting phylogenetic tree is shown in Figure 2. The phylogenetic analysis revealed that the six selected isolates were separated into three major phylogenetic groups corresponding to the genera *Salinivibrio*, *Halomonas*, and *Priestia*. Strains HK101 and HK116 clustered within the *Salinivibrio* clade; specifically, HK116 was positioned closest to *Salinivibrio kushneri* strain AL184, whereas HK101 clustered with *Salinivibrio siamensis* strain ND1-1 and *Salinivibrio sharmensis* strain BAG. Strain HK105 was grouped within the *Halomonas* clade and showed a close phylogenetic relationship with *Halomonas halophila* strain NBRC 102604. Within the *Priestia/Bacillaceae* lineage, the Gram-positive isolates showed distinct phylogenetic positions. HK125 clustered closely with *Priestia aryabhattai* strain B8W22 and HK142 grouped with *Priestia megaterium* strain ATCC 14581, whereas HK130 clustered with *Bacillus amyloliquefaciens* strain MPA 1034, forming a separate branch within the *Bacillaceae* lineage.

### 3.3. PHA Production of Selected Strains

The growth performance and PHA accumulation of the selected isolates cultivated on different carbon sources are summarized in Table 2. All strains were able to grow and accumulate PHA when cultivated on glucose, sucrose, glycerol, or the mixed-carbon strategy. DCW produced ranged from 1.30 ± 0.06 to 1.84 ± 0.09 g/L, depending on the strain and carbon source. PHA content varied widely across strains and substrates, ranging from 15.17 ± 0.81% to 48.46 ± 4.16% of DCW. Among the tested isolates, *Salinivibrio* sp. HK101 and *Priestia* sp. HK142 exhibited notably higher PHA accumulation, particularly when cultivated on glucose and sucrose. *Priestia* sp. HK142 showed the highest PHA content, reaching 48.46 ± 4.16 and 30.78 ± 1.84% DCW on glucose and sucrose, respectively. Similarly, HK101 accumulated high levels of PHA, with contents of 44.14 ± 3.28% and 47.37 ± 2.57% of DCW on glucose and sucrose, respectively.

GC–MS/MS analysis showed that PHA synthesized under single-carbon-source conditions consisted predominantly of 3HB, with molar fractions exceeding 99% in all strains. Incorporation of 3HV was detected only under the mixed carbon strategy, occurring in *Salinivibrio* sp. HK101 (6.4 mol%) and *Bacillus* sp. HK130 (3.2 mol%), while the remaining isolates produced homopolymeric PHB under the tested conditions. These results suggest that a mixed-carbon strategy may be more suitable for PHA production by *Salinivibrio* sp. HK101 and *Bacillus* sp. HK130, while single-carbon sources are sufficient for the remaining isolates.

Based on previous studies indicating the importance of the C/N ratio in PHA biosynthesis. The effect of the C/N ratio was evaluated at 20:1, 25:1, 30:1, and 35:1 (mol/mol). For *Salinivibrio* sp. HK101 and *Bacillus* sp. HK130, a mixed-carbon strategy was employed, with the proportions of glucose, 1,4-butanediol, and valeric acid kept constant while adjusting the total carbon input to achieve the target C/N ratios. The remaining strains (*Halomonas sp. HK105*, *Salinivibrio* sp. HK116, *Priestia* sp. HK125, and *Priestia* sp. HK142) were cultivated using their respective optimal single carbon sources identified in Table 2, with the C/N ratio varied accordingly.

As shown in Table 3, all selected isolates exhibited comparable cell growth across the tested C/N ratios, with DCW values ranging from 1.45 ± 0.04 to 2.09 ± 0.09 g/L. Variation in the C/N ratio resulted in distinct patterns of PHA accumulation among the isolates. Based on the C/N ratio yielding the highest PHA content, the strains were classified into two groups. *Halomonas* sp. HK105, *Bacillus* sp. HK130, and *Priestia* sp. HK142 showed maximum PHA accumulation at a C/N ratio of 25:1, with PHA contents ranging from 28.32 ± 2.01% to 50.72 ± 1.83% of DCW. In contrast, *Salinivibrio* sp. HK101, *Salinivibrio* sp. HK116, and *Priestia* sp. HK125 achieved their highest PHA contents at a C/N ratio of 30:1, ranging from 28.52 ± 1.91% to 42.07 ± 1.80% of DCW.

Among these, *Salinivibrio* sp. HK101 exhibited a pronounced increase in both PHA content and 3HV incorporation with increasing C/N ratio, reaching a maximum PHA content of 42.07% DCW at C/N 30:1, together with the highest 3HV molar fraction (6.4 mol%) observed for this strain. *Bacillus* sp. HK130 also showed the ability to incorporate 3HV, but at consistently lower molar fractions, ranging from 1.9 to 4.4 mol% across the tested C/N ratios. In contrast, HK142 consistently showed the highest PHA accumulation among all isolates, with PHA contents exceeding 48% DCW at C/N ratios of 25:1, 30:1, and 35:1, and reaching a maximum of 50.72% DCW at C/N 25:1.

### 3.4. PHA Characterization of Salinivibrio sp. HK101 and Priestia sp. HK142 Strains

Based on the above results, *Salinivibrio* sp. HK101 and *Priestia* sp. HK142, representing a copolymer-producing strain and a high-PHB-producing strain, respectively, were selected for further evaluation of the structural characteristics of the produced PHA.

**Figure 3:**
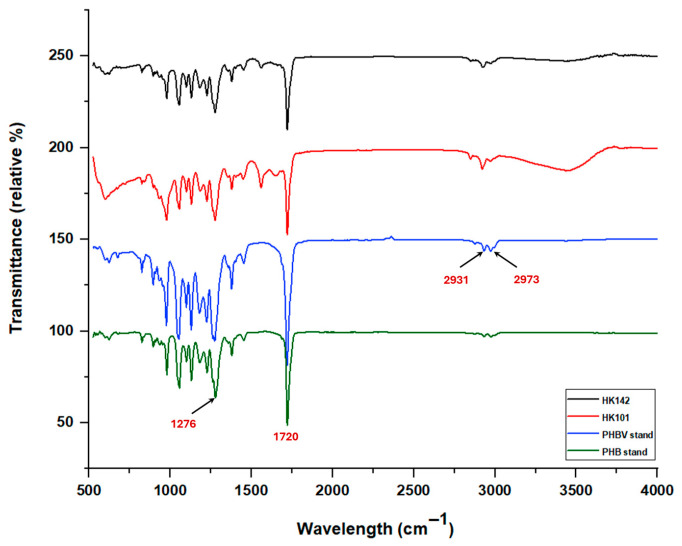
FTIR spectra of purified PHA produced by strains *Salinivibrio* sp. HK101 and *Priestia* sp. HK142, in comparison with PHBV and PHB standards.

As shown in Figure 3, the FTIR spectra of the purified polymers produced by *Salinivibrio* sp. HK101 and HK142 exhibited characteristic absorption bands of PHA and were compared with those of PHB and PHBV standards. All spectra showed a strong band at approximately 1720 cm^−1^, corresponding to the ester carbonyl (C=O) stretching vibration. The FTIR spectrum of *Priestia* sp. HK142 closely overlapped with that of the PHB standard over the entire spectral range, particularly in the carbonyl region and the fingerprint region (1300–1000 cm^−1^). In contrast, the spectrum of *Salinivibrio* sp. HK101 showed greater similarity to the PHBV standard, with noticeable differences observed in the C–H stretching region (3000–2800 cm^−1^). Absorption bands at approximately 2931 and 2973 cm^−1^, assigned to C–H stretching vibrations of alkyl groups, were more pronounced in the spectra of *Salinivibrio* sp. HK101 and the PHBV standard than in *Priestia* sp. HK142 and the PHB standard. An absorption band at around 1276 cm^−1^, attributed to the C–O stretching vibration of ester linkages, was observed in the spectra of both produced polymers as well as the corresponding standards.

**Figure 4:**
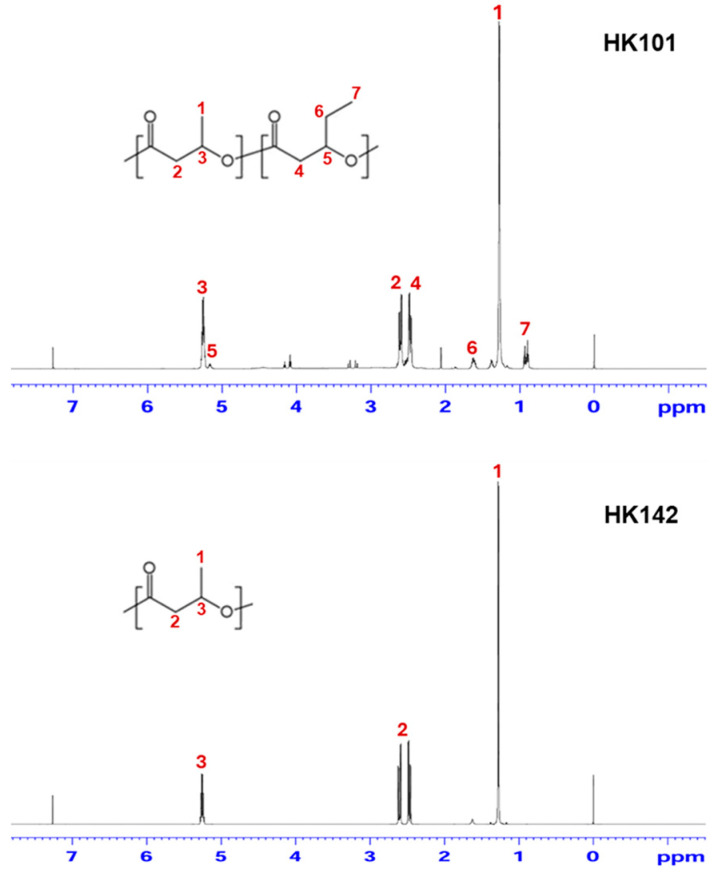
^1^H-NMR spectra of PHA polymers produced by *Salinivibrio* sp. HK101 and *Priestia* sp. HK142 strains.

To provide additional molecular-level information on the polymer structure, ^1^H-NMR analysis was subsequently performed. As shown in Figure 4, the ^1^H-NMR spectrum of the polymer produced by *Priestia* sp. HK142 exhibited characteristic proton resonances of PHB. A dominant signal at approximately 1.2–1.3 ppm was observed, corresponding to the methyl protons (–CH_3_) of the 3HB unit, accompanied by signals at around 2.4–2.6 ppm and 5.2 ppm, which were assigned to the methylene (–CH_2_–) and methine (–CH–) protons of the 3HB backbone, respectively. No additional proton signals attributable to other monomer units were detected in the *Priestia* sp. HK142 spectrum.

In contrast, the ^1^H-NMR spectrum of the polymer produced by *Salinivibrio* sp. HK101 showed, in addition to the characteristic proton signals of 3HB units, extra resonances associated with the presence of 3HV units. Specifically, signals appearing in the 0.8–0.9 ppm region were assigned to the terminal methyl protons of the 3HV side chain, while additional methylene proton signals were observed in the 1.6–1.7 ppm range. These signals coexisted with those corresponding to 3HB units in the *Salinivibrio* sp. HK101 spectrum.

## 4. Discussion

The present study investigated the microbial diversity of the Hon Khoi saltern in Khanh Hoa Province, Vietnam, and revealed that this environment contains a diverse population of halotolerant bacteria capable of synthesizing PHAs. A total of 37 halotolerant bacterial isolates were obtained from salt field samples, among which six strains, *Salinivibrio* sp. HK101 and HK116, *Halomonas* sp. HK105, *Priestia* sp. HK125 and HK142, and *Bacillus* sp. *Bacillus* sp. HK130, demonstrated intracellular polyester accumulation. The Hon Khoi saltern is reported to be one of the largest traditional salt production areas in Vietnam, with an estimated annual production of approximately 30,000 tons of salt. This hypersaline environment has high salinity, intense sunlight, and limited nutrient availability. These conditions favor the selection of microorganisms with specialized physiological adaptations. However, this environment remains largely unexplored for halotolerant bacteria that are capable of producing valuable biopolymers like PHAs. Therefore, this work represents one of the first systematic investigations of PHA-producing halotolerant bacteria isolated from the Hon Khoi saltern, contributing to the understanding of microbial resources in Vietnamese saltern ecosystems and highlighting their potential for biotechnological applications.

In this study, intracellular polyester accumulation was detected using staining methods using the Sudan Black B (lyophilic) and Nile Blue A (fluorescent) dyes. These approaches are commonly recognized as standard preliminary screening methods for identifying PHA-producing bacteria [28]. Sudan Black B staining facilitates the visualization of intracellular lipid inclusions [29], whereas Nile Blue A selectively stains PHA granules, exhibiting distinctive fluorescence under UV illumination [21]. TEM, FTIR, and GC–MS/MS further validate the presence and morphology of intracellular PHA granules. These combined methods have been widely used in recent studies to accurately identify PHA-producing microorganisms isolated from various environments. For example, Ibrahim et al. (2025) reported the application of Sudan Black B staining to detect PHA accumulation in *Bacillus australimaris* isolated from soil samples, subsequently employing FTIR analysis to verify the chemical structure of the polymer [30]. In a similar study, Mohammed et al. (2019) used Sudan Black B and Nile Blue A staining to screen approximately 200 bacterial isolates from a plastic waste landfill, identifying *Bacillus* sp. BPPI-14 and *Bacillus* sp. BPPI-19 as a potential PHA producer [31]. The presence of PHA in these strains was subsequently verified by FTIR spectroscopy. In addition to staining-based screening methods, Kanavaki et al. (2021) identified PHB production in *Pseudomonas* sp. phDV1 using TEM to visualize intracellular granules, while the polymer structure was confirmed through FTIR, NMR, and MALDI-TOF/TOF analyses [22]. Therefore, the application of multiple complementary techniques in this study provides reliable preliminary evidence for intracellular polyester accumulation in the isolates.

Phylogenetic identification based on 16S rRNA gene sequencing revealed that the six PHA-producing isolates are classified into three genera: *Salinivibrio*, *Halomonas*, *Bacillus,* and *Priestia*. These genera are known to include halotolerant or moderately halophilic bacteria commonly found in saline and marine environments. The genus *Halomonas* is widely recognized as an important group of halophilic bacteria with significant biotechnological potential, especially in the production of PHAs. Numerous *Halomonas* strains have been documented to synthesize PHB and related copolymers while tolerating high salinity (0.5–25% NaCl) and high temperatures (25–40 °C) and utilizing diverse carbon substrates [32,33,34,35]. These physiological traits of *Halomonas* spp. render them particularly attractive for industrial PHA production because cultivation under saline conditions reduces the risk of contamination and may simplify downstream processing [36,37,38]. Species of the genus *Salinivibrio* are generally isolated from hypersaline environments and fermented foods. For instance, *Salinivibrio siamensis* was initially identified in fermented fish in Thailand and represents a typical moderately halophilic species capable of thriving in saline environments (1.0–20% NaCl and 10–50 °C) [39,40,41]. Several *Salinivibrio* isolates, including *Salinivibrio* sp. TGB4, *Salinivibrio* sp. TGB19, and *Salinivibrio* sp. TGB10, have been found to be capable of producing biopolymer PHB using acetate and starch as carbon sources [42,43]. Many species within the genus *Bacillus* are halotolerant and are commonly found in saline soils and coastal environments. Several studies have reported that different *Bacillus* species can tolerate NaCl concentrations up to approximately 10–15% and grow within mesophilic temperature ranges of about 25–45 °C [44,45]. Several *Bacillus* species have been reported to accumulate intracellular PHA under suitable culture conditions [46,47]. These physiological traits enable *Bacillus* species to survive and grow in environments with relatively high salinity. Members of the genus *Priestia* are generally considered moderately halotolerant bacteria. Several studies have reported that *Priestia* strains can grow over a relatively broad temperature range (approximately 20–45 °C) and tolerate NaCl concentrations up to about 6–10% [48,49].

The physiological characterization of the isolates revealed a broad tolerance to salinity and temperature. Most strains were able to grow in media containing 3–10% NaCl, whereas only the *Halomonas* sp. *HK105* isolate demonstrated tolerance to 15% NaCl. This observation is consistent with previous reports indicating that members of the genus *Halomonas* generally exhibit higher salt tolerance compared with other genera, such as *Salinivibrio*, *Bacillus*, and *Priestia*, which are typically classified as moderately halophilic or halotolerant bacteria. For instance, *Salinivibrio* species exhibit optimal growth at 7.0–7.5% NaCl and 37 °C; *Bacillus* strains thrive within a range of 5–10% NaCl at approximately 35 °C; and *Priestia megaterium* shows tolerance to salinity levels of up to 9% NaCl under comparable mesophilic conditions [40,41,48,50]. The isolates also exhibited robust growth across a relatively wide temperature range from 25 to 45 °C. This tolerance presumably indicates their adaptability to the dynamic conditions of saltern ecosystems, where salinity and temperature fluctuate during evaporation cycles. These characteristics may also enhance their suitability for biotechnological applications, including the production of biodegradable polymers or biofuels, which are increasingly important in sustainable development [51].

Among the tested isolates, *Salinivibrio* sp. HK101 and *Priestia* sp. HK142 showed the highest capacity for PHA accumulation. The highest PHA accumulation was achieved by *Priestia* sp. HK142 under optimized cultivation conditions at a C/N ratio of 25:1, with a PHA concentration of 2.09 ± 0.09 g/L, resulting in 50.72 ± 1.83% of DCW. Several species within the genus *Priestia*, particularly *P. megaterium*, have been reported to accumulate PHB at concentrations ranging from approximately 1.5 to 3.1 g/L, with intracellular polymer contents typically reaching about 50–63% of DCW, depending on the strain and cultivation conditions [47,52,53,54]. The values of *Priestia* sp. HK142 are within the range reported for previously characterized *Priestia* strains, indicating that this isolate exhibits a comparable PHA production capacity and may represent a promising candidate for further optimization.

*Salinivibrio* sp. HK101 reached its highest PHA content at a C/N ratio of 30:1, which corresponds to 42.07 ± 1.8% of DCW and 1.45 ± 0.04 g/L. Members of the genus *Salinivibrio* have been reported to accumulate PHB under nutrient-limited conditions. The intracellular polymer contents of these members typically range from approximately 33.5% to 83.8% of DCW (PHB concentration 0.23–8.5 g/L) depending on strain and cultivation strategy [43,55]. Although the PHA content of strain *Salinivibrio* sp. HK101 is relatively lower than that of highly optimized *Salinivibrio* cultures, it is within the range that is commonly observed for newly isolated wild-type strains.

In addition, nitrogen limitation combined with an excess carbon supply is a well-known trigger for PHA accumulation in many bacteria [33,56]. Under nutrient-limited conditions, cells redirect surplus carbon and store it in the form of intracellular polymers. The C/N ratios identified in this study are consistent with previous reports on PHA production in halophilic bacteria, where optimal ratios commonly range from 20:1 to 40:1 depending on the strain and carbon substrate [56,57]. Elevated C/N ratios have been reported to exert an inhibitory effect on PHA storage capacity in microbial communities [57]. The combination of salt tolerance, broad substrate utilization, and relatively high PHA accumulation observed in strains *Salinivibrio* sp. HK101 and *Priestia* sp. HK142 highlights their potential as candidates for further optimization and scale-up in saline fermentation systems.

In conclusion, the findings of this study demonstrate that the Hon Khoi saltern represents a promising source of halotolerant bacteria capable of producing PHAs. Further work will be required to characterize the biosynthetic pathways and fermentation performance of these isolates on larger scales. Genome sequencing and metabolic analysis may provide insights into the genetic determinants underlying their salt tolerance and PHA biosynthesis capabilities. In addition, future studies should explore process optimization and the use of low-cost substrates to evaluate the feasibility of industrial PHA production using these halotolerant strains.

## Figures and Tables

**Table 1:** Morphological, physiological, and biochemical characteristics of the six selected halotolerant bacterial isolates.

Characteristic	HK101	HK105	HK116	HK125	HK130	HK142
Colony morphology	Round, smooth, convex, opaque, cream colonies (2.0–2.5 mm).	Round, smooth, convex, opaque, yellow colonies (0.5–1.0 mm)	Round, smooth, convex, opaque, light cream colonies (0.8–1.2 mm)	Round, irregular-surfaced, opaque, creamy white colonies (2.0–3.0 mm).	Round, irregular-surfaced, opaque, grayish-white colonies (2.0–2.6 mm)	Round, undulate margin, opaque, cream colonies (2.0–2.5 mm)
Cell morphology	Curved rods	Rods	Curved rod	Rods	Rods	Rods
Motility	+	+	+	+	+	+
Spore formation	−	−	−	+	+	+
Gram staining	−	−	−	+	+	+
Oxidase	+	+	+	−	+	+
Catalase	+	+	+	+	+	±
Voges-Proskauer	+	−	+	+	+	−
Growth at 0% NaCl	−	−	−	−	+	−
3% NaCl	+	+	+	+	+	+
5% NaCl	+	+	+	+	+	+
10% NaCl	+	+	−	+	−	−
15% NaCl	−	+	−	+	−	−
Growth at 25 °C	+	+	+	+	+	+
30 °C	+	+	+	+	+	+
37 °C	+	+	+	+	+	+
40 °C	+	+	+	+	+	+
45 °C	+	−	+	±	+	+
Sugar fermentation:						
D-glucose	+	+	+	+	+	+
Sucrose	+	−	+	+	+	+
Maltose	+	+	+	+	+	+
D-galactose	−	+	−	+	+	+
L-arabinose	−	+	−	±	−	+
D-xylose	+	+	−	−	−	+
D-mannose	+	+	+	+	+	−
D-mannitol	+	−	+	±	+	+
Hydrolysis:						
Soluble starch	+	−	+	+	+	+
Casein	+	−	−	+	+	+
CMC	±	−	+	+	−	+

(+) positive; (−) negative; (±) weakly positive. Colony morphology was recorded after incubation on high-salt LB agar supplemented with 3% (*w*/*v*) NaCl at 37 °C for 24–48 h. Growth under different NaCl concentrations and temperatures was assessed after 48 h of incubation.

**Table 2:** Growth and PHA accumulation of selected isolates cultivated on different carbon sources.

Strain	Carbon Source	DCW (g/L)	PHA Content (% DCW) **	PHA Component (%mol) **	
				3HB	3HV
*Salinivibrio* sp. HK101	Glucose	1.69 ± 0.07	44.14 ± 3.28	100	0
	Sucrose	1.71 ± 0.08	47.37 ± 2.57	100	0
	Glycerol	1.59 ± 0.05	30.82 ± 1.59	100	0
	Mixture *	1.45 ± 0.04	42.07 ± 1.80	93.6	6.4
*Halomonas* sp. HK105	Glucose	1.57 ± 0.07	27.77 ± 3.90	100	0
	Sucrose	1.61 ± 0.08	28.32 ± 2.01	100	0
	Glycerol	1.53 ± 0.06	28.10 ± 1.71	100	0
	Mixture *	1.30 ± 0.06	32.31 ± 2.06	100	0
*Salinivibrio* sp. HK116	Glucose	1.59 ± 0.09	26.33 ± 2.42	100	0
	Sucrose	1.66 ± 0.07	28.52 ± 1.91	100	0
	Glycerol	1.45 ± 0.04	15.17 ± 0.81	100	0
	Mixture *	1.50 ± 0.05	26.67 ± 2.12	100	0
*Priestia* sp. HK125	Glucose	1.64 ± 0.04	31.65 ± 2.08	100	0
	Sucrose	1.59 ± 0.07	30.78 ± 1.47	100	0
	Glycerol	1.56 ± 0.08	26.92 ± 1.88	100	0
	Mixture *	1.47 ± 0.08	31.97 ± 2.42	100	0
*Bacillus* sp. HK130	Glucose	1.61 ± 0.06	30.07 ± 2.41	100	0
	Sucrose	1.50 ± 0.07	24.70 ± 1.71	100	0
	Glycerol	1.42 ± 0.08	25.35 ± 2.01	100	0
	Mixture *	1.48 ± 0.07	22.97 ± 1.28	96.8	3.2
*Priestia* sp. HK142	Glucose	1.84 ± 0.09	48.45 ± 4.16	100	0
	Sucrose	1.72 ± 0.07	30.78 ± 1.84	100	0
	Glycerol	1.62 ± 0.09	24.07 ± 1.82	100	0
	Mixture *	1.59 ± 0.08	26.42 ± 1.74	100	0

* Mixture indicates a mixed carbon strategy in which glucose (20 g/L) and 1,4-butanediol (9 g/L) were supplied at the beginning of cultivation, followed by the addition of valeric acid (1 g/L) after 24 h of fermentation. ** PHA content (% DCW) and the molar fractions of 3HB and 3HV were calculated from GC–MS/MS chromatograms following acidic methanolysis.

**Table 3:** Growth and PHA accumulation of selected isolates cultivated at different carbon-to-nitrogen (C/N) ratios.

Strain	C/N Ratio (mol/mol)	DCW (g/L)	PHA Content (% DCW) *	PHA Component (%mol) *	
				3HB	3HV
*Salinivibrio* sp. HK101	20:1	1.78 ± 0.05	23.60 ± 1.30	97.3	2.7
	25:1	1.85 ± 0.06	35.68 ± 1.99	94.2	5.8
	30:1	1.45 ± 0.04	42.07 ± 1.80	93.6	6.4
	35:1	1.52 ± 0.07	30.26 ± 1.75	95.8	4.2
*Halomonas* sp. HK105	20:1	1.84 ± 0.06	14.13 ± 0.71	100	0
	25:1	1.75 ± 0.07	26.06 ± 2.01	100	0
	30:1	1.61 ± 0.08	28.32 ± 2.01	100	0
	35:1	1.62 ± 0.07	20.99 ± 1.33	100	0
*Salinivibrio* sp. HK116	20:1	1.88 ± 0.06	17.55 ± 0.77	100	0
	25:1	1.82 ± 0.06	26.65 ± 1.87	100	0
	30:1	1.66 ± 0.07	28.52 ± 1.91	100	0
	35:1	1.66 ± 0.08	28.43 ± 1.66	100	0
*Priestia* sp. HK125	20:1	1.92 ± 0.04	24.48 ± 1.16	100	0
	25:1	1.89 ± 0.06	28.10 ± 1.82	100	0
	30:1	1.64 ± 0.04	31.65 ± 2.08	100	0
	35:1	1.73 ± 0.09	29.94 ± 1.92	100	0
*Bacillus* sp. HK130	20:1	1.68 ± 0.05	16.67 ± 0.78	98.1	1.9
	25:1	1.68 ± 0.08	29.17 ± 1.83	95.6	4.4
	30:1	1.48 ± 0.07	22.97 ± 1.28	96.8	3.2
	35:1	1.71 ± 0.07	19.88 ± 0.97	97.3	2.7
*Priestia* sp. HK142	20:1	1.96 ± 0.07	29.08 ± 1.46	100	0
	25:1	2.09 ± 0.09	50.72 ± 1.83	100	0
	30:1	1.84 ± 0.09	48.45 ± 4.16	100	0
	35:1	1.77 ± 0.06	50.28 ± 1.97	100	0

* PHA content (% DCW) and the molar fractions of 3HB and 3HV were calculated from GC–MS/MS chromatograms.

## Data Availability

The original contributions presented in this study are included in the article. Further inquiries can be directed to the corresponding author.

## Abbreviations

PHA	Polyhydroxyalkanoates
3HB	3-Hydroxybutyrate
3HV	3-Hydroxyvalerate
PHB	Poly(3-hydroxybutyrate)
PHBV	Poly(3-hydroxybutyrate-co-3-hydroxyvalerate)
TEM	Transmission Electron Microscopy
GC–MS/MS	Gas Chromatography–Tandem Mass Spectrometry
FTIR	Fourier Transform Infrared Spectroscopy
DCW	Dry Cell Weight
CMC	Carboxymethyl Cellulose
